# Abnormal Behavior Episodes Associated With Zonisamide in Three Dogs: A Case Report

**DOI:** 10.3389/fvets.2021.763822

**Published:** 2021-10-29

**Authors:** Shinichi Kanazono, Masayasu Ukai, Akira Hiramoto

**Affiliations:** ^1^Neurology and Neurosurgery Service, Veterinary Specialists & Emergency Center, Kawaguchi, Japan; ^2^Saitama Animal Medical Center, Iruma, Japan; ^3^Department of Clinical Studies, Ontario Veterinary College, University of Guelph, Guelph, ON, Canada

**Keywords:** episodic abnormal behavior, psychiatric adverse effect, anti-seizure drug, zonisamide, dog, epilepsy

## Abstract

Psychiatric adverse effect associated with anti-seizure drugs has been well-recognized in human medicine. This case report describes three dogs with presumptive idiopathic epilepsy presented for abnormal behavior episodes. Abnormal behavior episodes included sudden rage and aggression to the family members, insomnia, restlessness, and/or constant attention-seeking behavior. MRI study and cerebrospinal fluid analysis in two dogs were unremarkable. The abnormal behavior episodes deteriorated along with gradual dose increment of zonisamide and these episodes almost completely disappeared within 5 days after discontinuation of zonisamide. The exact same episodes relapsed within days after re-administration of zonisamide and disappeared again shortly after discontinuation of zonisamide. Dose adjustments of other anti-seizure medications in case 2 did not result in significant changes in these behavior episodes. Although psychiatric adverse effects including aggressive behavior associated with zonisamide are widely recognized in humans, this is the first report in dogs in the clinical setting.

## Introduction

Zonisamide (ZNS) is a benzisoxazole derivative with a non-arylamine sulfonamide group (1) and is chemically unrelated to other anti-seizure drugs (ASDs) used in veterinary medicine. With limited available evidence supporting its efficacy in the veterinary field, this medication has been widely used for anti-seizure purpose in dogs and in humans (2–7). In addition to its anti-seizure potency, ZNS may be efficacious in treating various human neurological and psychiatric diseases including migraine, neuropathic pain, essential tremor, and Parkinson's disease (8–12).

The recommended dose and monitoring strategy are as follows: oral starting dose at 3–7 mg/kg every 12 hours (q12h) or 7–10 mg/kg q12h in dogs with co-administered hepatic microsomal enzymes inducers such as phenobarbital, serum concentrations should be aimed between 10 and 40 mg/L according to the human target range, and the serum concentration measurements should be performed at least 1 week after treatment initiation or dosage adjustment given the approximate elimination half-life of 15 h (6, 7). Reported adverse effects of ZNS in dogs in the clinical setting include sedation, generalized ataxia, vomiting, inappetence, and a few idiosyncratic reactions such as cutaneous reactions (13, 14), acute hepatopathy (15, 16), and renal tubular acidosis (17). In addition, aggression has been reported in a research setting investigating chronic toxicity of ZNS in dogs (18). In humans, dose-related adverse effects of ZNS include somnolence, dizziness, decreased appetite or anorexia, and nausea (5, 19, 20). Other reported adverse effects include fatigue, headache, psychiatric symptoms, cognitive disturbances, diplopia, weight loss, diarrhea, ataxia, oligohydrosis, urolithiasis, and rash (19–24). Here, we report three dogs with abnormal behavior episodes associated with ZNS.

## Case Presentation

### Case 1

An 11-year-old male castrated golden retriever that weighed 29.1 kg was evaluated at Saitama Animal Medical Center for recently increasing frequency of the generalized tonic–clonic seizures (GTCS). The owner described this dog had exhibited epileptic episodes once every 2–3 months since he was 8 years old and his episodes were becoming more frequent to once per month over the past 8 months.

Prior to the referral, this dog had been managed with ZNS starting at 2.5 mg/kg per os (PO) q12h then gradually increased in the dose up to 5 mg/kg q12h with the trough blood concentration at 21.2 μg/ml over the 15 months. No other ASDs such as phenobarbital or potassium bromide, listed as first-line ASDs in dogs, were prescribed. Zonisamide was further increased to 7.5 mg/kg q12h due to a few events of cluster of seizures. Two more months later, levetiracetam (LEV) at 17 mg/kg PO q12h was added on ZNS as the dog experienced another episode of cluster of seizures.

Upon the initial visit, the physical and neurological examinations were overall unremarkable other than severe degenerative joint condition in his bilateral elbow, coxofemoral, and stifle joints. Complete blood count (CBC), serum biochemical analysis, and urinalysis were within the reference intervals. Our initial plan was to gradually increase ZNS dosage as needed and to maintain LEV at the same dose. Firocoxib was also prescribed for the joint condition. Zonisamide was prescribed with the gradual dose increment plan potentially up to 13.7 mg/kg q12h if needed based on his seizure frequency. A 2-week telephone follow-up revealed this dog was exhibiting unusual behavior episodes of abrupt barking during sleep without obvious external stimuli. This dog had no pre-existing abnormal behavior problems before and no seizure episodes were witnessed in association with these behavior episodes. At this point, ZNS dose was 10 mg/kg q12h. Another week later, the owner reported the increasing frequency and the change in the episodic abnormal behavior. According to the owner, this dog suddenly stood up during the sleep to bite his tail or blanket for a minute. When the owner called him during the abnormal behavior episode, the dog was able to come out of it and went back to himself. Ten more days later, the owner described that abnormal behavior episodes were deteriorating in the following aspects: increasing in frequency to daily events, not limited to during the sleep, and deteriorating in its severity to bite the owner on her arm severe enough to visit an emergency hospital. The frequency of his GTCS decreased after increment of ZNS up to 13.7 mg/kg q12h while the abnormal behavior episodes increased in its frequency further to over 10 times a day, including abrupt growling at or attempts to bite the family members when they came into his sight while awake (Supplementary Video 1). An MRI and cerebrospinal fluid analysis were both unremarkable. Phenobarbital (PB) at 1.5 mg/kg q12h was added at this point concerning these abnormal behavior episodes as an atypical manifestation of sensory epileptic events. The owner reported 7 days later that no GTCS was witnessed while the episodic abnormal behavior remained at the same intensity and frequency. Zonisamide was discontinued abruptly and PB was increased to 2 mg/kg q12h. Seven days after the discontinuation of ZNS, the owner reported those frequent abnormal behavior episodes almost completely disappeared within 5 days after discontinuation of ZNS except for occasional gesture curling up the upper lip during the sleep for a few seconds. Within another week, the abnormal behavior episodes completely disappeared. Our recommendation of reintroducing ZNS for confirmation of direct association between the aggressive behavior and ZNS was rejected by the owner for safety concern of the family members. This dog was managed well with PB (2 mg/kg q12h) and LEV (20 mg/kg q8h) with no observable epileptic or aggressive behavior episodes for 13 months until this dog died of an unrelated condition.

### Case 2

A 6-year-old female spayed miniature poodle weighing 5.05 kg was referred to Veterinary Specialists and Emergency Center for episodic aggression. The aggression was noted in the absence of precipitating causes or environmental triggers, occurring nightly predominantly toward females in the household. The aggressive behavior toward the female child was particularly noteworthy, biting her as she slept. Episodic aggression toward the adult female occurred while the dog is being picked up and held (Supplementary Video 2). The episodic aggression lasted up to 30 s in duration, returning back immediately following. Episodes showed no response to acepromazine (unknown dose or route).

Prior to referral, this dog had been on ZNS at the dose of 10 mg/kg PO q12h for presumptive idiopathic epilepsy over the past 3 years and epilepsy had been extremely well-controlled with no seizure episodes witnessed. Other than episodic aggression, the dog remained healthy and happy. No significant changes in the environment were reported.

Upon the initial visit, the physical and neurological examinations were overall unremarkable. CBC, serum biochemical analysis, and urinalysis were within the reference intervals. Serum level of ZNS was 47.8 μg/ml. MRI and cerebrospinal fluid analysis were rejected by the owner given the concerns associated with general anesthesia and long history of presumptive idiopathic epilepsy.

Due to serious detrimental impact on the family, ZNS was discontinued abruptly as a therapeutic trial with our concern of possible association between the episodic aggression and ZNS. Phenobarbital 2.5 mg/kg orally q12h was prescribed without loading. The nightly episodic aggression completely disappeared within 5 days after discontinuation of ZNS. This dog had an episode of cluster seizures. After failing to control daily epileptic episodes with PB, LEV, and potassium bromide over the next 10 days, ZNS at 50% reduced dose (5 mg/kg PO q12h) was resumed to confirm the direct association between ZNS and the aggressive episodes as well as to manage seizure episodes. Daily seizure episodes resolved after ZNS was added. While the dog remained herself over the next 3 days during hospitalization after reintroduction of ZNS, the exact same episodes relapsed on the night after discharge. The aggressive episodes deteriorated day by day over the next 5 days until ZNS dose was reduced to 5 mg/kg PO in the morning and 2.5 mg/kg PO in the evening. Seven days later, the owner reported episodic aggression was improved by 40% compared with that prior to dose reduction. No seizure episodes were recognized. Over the next 6 months, several dose adjustments of ASDs were made. Episodic aggression deteriorated with minor dose increment of ZNS and improved with minor dose reduction.

**Figure 1:**
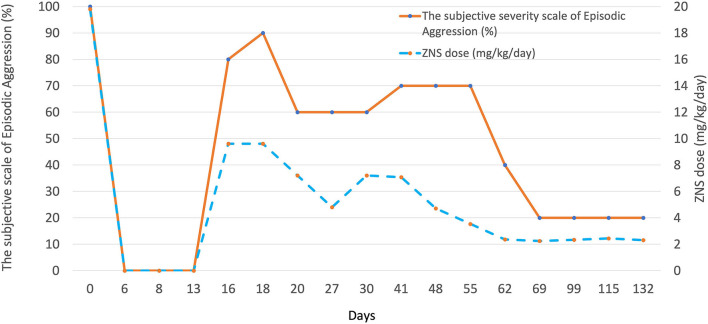
A graph illustrating the association between the dose of ZNS and the severity of episodic aggression in case 2. Multiple dose adjustments resulted in significant changes in the severity of the abnormal behavior episodes in this case. The severity of episodic aggression was determined based on the client's oral description at each follow-up; 100% at the initial presentation.

The severity of the episodic aggression was scaled as 100% at the initial appointment and the sequential change in the severity was evaluated by the client and provided at each follow-up appointment in the gross scale of 0–100%. Chronological changes in episodic aggression in association with dose adjustments of ZNS are summarized in Figure 1. The severity and frequency of the episodic aggression showed proportional relationship with the dose of ZNS. No similar episodic aggression was witnessed in association with dose adjustments or addition of other ASDs including PB, potassium bromide, and LEV.

### Case 3

A 10-year-old male castrated miniature poodle was referred to Veterinary Specialists and Emergency Center for recent onset of generalized seizure episodes, decreased appetite, and abnormal behavior.

This dog had clinical onset of generalized seizure 12 days prior to referral. The seizure episode was described as sudden running fit with falling and defecation, followed by 1–2-min duration of opisthotonus, clonic motor activities, and copious amount of ptyalism. Within 24 h after ZNS was commenced at the dose of 5 mg/kg PO q12h, this dog started acting abnormal. His behavior was characterized by insomnia, agitation, constant attention-seeking behavior through the night, restlessness, and excessive reaction to the external stimuli. Routine bloodwork consisting of CBC and serum biochemical analysis were within the reference intervals. The dose of ZNS was increased to 6.6 mg/kg PO q12h by a referring veterinarian. MRI, cerebrospinal fluid analysis, and fasted and postprandial total bile acid analyses were within normal limits. Addition of LEV (27 mg/kg PO q8h) and glycerin (5 ml/head PO q8h) did not alter the abnormal behavior and decreased appetite.

Upon the initial visit, the physical and neurological examinations were overall unremarkable. Given the history and unremarkable clinicopathological analyses results, ZNS dose was reduced to 4.5 mg/kg PO q12h without any changes in LEV and glycerine to avoid multiple variables at one time for assessing the clinical response to the medication adjustment. The owner reported that significant improvements were recognized within 24 h after the dose reduction and no seizure episodes were witnessed. The dose of ZNS was further decreased to 2.3 mg/kg PO q12h without any changes in LEV and glycerine for another 3 days then completely discontinued. Seven days after complete discontinuation of ZNS, abnormal behavior episode completely disappeared, and the appetite was recovered by 80% according to the owner. However, sudden jerky movement of the upper body was increasingly noticed along with tapering off process of ZNS. Therefore, the dose of LEV was increased to 40 mg/kg PO q8h, resulting in significant improvement of the jerky movements without relapse in the abnormal behavior.

Seven days after the dose increment of LEV, ZNS was re-introduced at 6.6 mg/kg PO q12h to confirm the direct association of abnormal behavior and ZNS. The owner reported the same abnormal behavior episodes relapsed within 12 h after re-introduction and gradually deteriorated over the next 3 days. Zonisamide was abruptly discontinued 4 days after re-introduction, which resulted in resolution of the abnormal behavior episodes within 24 h. No other modifications in other medications were made during this ZNS re-introduction trial.

## Discussion

This case series reports the occurrence of reversible behavior changes associated with zonisamide in three dogs with no pre-existing behavior problems prior to administration of zonisamide. Among various ASDs, ZNS has been frequently used in veterinary practice with several adverse effects being reported (2, 7, 13–17, 25–32). Reported type 1 adverse effects include sedation, vomiting, loss of appetite, and ataxia (2, 7, 25, 28). Reported type 2 adverse effects include two cases of acute toxic liver injury, three cases with dermatologic lesions, and one case of renal tubular acidosis (13–17). Other potential type II adverse effects include one case with keratoconjunctivitis sicca and one case with polyarthropathy, although the direct relationship was not confirmed in these cases (7). As for type 3 adverse effects, the possibility of affecting thyroid function (especially decrease in total T4) and the changes in blood chemistry profile within the reference range, including the elevation of ALP and Ca and the decrease of total protein and albumin compared with those prior to the administration of ZNS, were observed (7). Adverse effects classified as type 4 have not been reported (7). Recently, ZNS-related anticonvulsant hypersensitivity syndrome was reported in cats (27). However, aside from a previous report by Walker et al. in the research setting where three male dogs showed aggression while they received 75 mg/kg/day of ZNS for 52 weeks achieving the serum ZNS level around 80–120 μg/ml, (18) abnormal behavior episodes as we experienced have not been reported in the clinical veterinary setting to the best of our knowledge.

In human medicine, there were several reports of psychiatric and behavioral side effects associated with ZNS (1, 24, 33–36). A review article reported the most common adverse effects pertained to the CNS were ataxia, dizziness, somnolence, agitation, and anorexia (36). Another report described that psychiatric adverse events (PAEs) and cognitive adverse events (CAEs) were the most frequently identified reasons for terminating ZNS therapy in 433 epileptic patients who received ZNS (24). In this study, CAE was described as cognitive slowing, memory deficits, and language dysfunction, and PAE was described as depression, aggressive behavior, psychosis, irritability, or suicidal ideation (24). The incidence of PAE severe enough to result in discontinuation of ZNS was 6.9%; the incidence of CAE resulting in discontinuation of ZNS was 5.8%. These patients improved shortly after ZNS was discontinued. Changes in brain serotonin and dopamine levels are considered as a mechanism of ZNS-induced PAE (24, 35, 37) and human patients with a history of psychiatric disorder had significantly greater risk of developing PAE associated with ASDs including ZNS (24, 36, 38, 39). It was also interesting that the average maximum ZNS serum concentration in patients who discontinued ZNS attributable to PAE or CAE was significantly lower than maximum ZNS concentration of control group in the aforementioned study (24). This may suggest that PAE or CAE are not necessarily associated with higher serum concentration of ZNS than the reference range. While most of the human patients developed PAE or CAE in the first 3 months of exposure to ZNS and resulted in discontinuation of ZNS within 5 months, some human patients developed PAE or CAE after many months after ZNS treatment was started, often in association with late dose increment of ZNS (24).

Our cases showed clinical apparent direct association with ZNS and correlation with dose adjustment in case 1 and case 2. This suggests that ZNS-related PAE may be a dose-dependent adverse effect that could be overlapped with the therapeutic reference range in predisposed patients. Although therapeutic and toxic range of ZNS in dogs has not been well-established yet, serum concentration at 47.8 μg/ml in our case 2 could be considered in high end or above the therapeutic range (6, 7, 25, 28). The aggressive behavior resolved almost completely within 5 days after the abrupt cessation of ZNS in our cases. This may also support the possible dose-dependent nature of the aggressive episodes, given the reported elimination half-life of ZNS in dogs being approximately 15 h (6, 26).

In two recent reports on human medicine, LEV had the highest PAE risk (15.7–16.2%), which was significantly higher compared with those with other ASDs (38, 39). Similarly, a case series study described abnormal behavior episodes in dogs receiving LEV (40). Interestingly, 51.2% of LEV-attributed PAE symptoms in humans resolved with dose decrement, whereas only 15.4% patients of ZNS-attributed PAE symptoms resolved with dose decrement and the rest (85.6%) of ZNS-attributed PAE symptoms required complete cessation of ZNS (38). In our cases 1 and 2, ZNS was discontinued abruptly due to serious impact of the aggressive behavior episodes to the family and human data according to the aforementioned report (38).

Limitations of this report reside in the small number of cases, the retrospective nature, and difficulty in proving the direct association of abnormal behavior episodes and ZNS or in ruling out other potentially contributing conditions. Our case 2 did not have MRI or cerebrospinal fluid analysis to rule out other intracranial structural conditions, which was clinically considered relatively unlikely given stable condition through a long follow-up duration of this case. Nonetheless, the proportional association between ZNS dose and the severity of the clinical signs was shown with multiple dose adjustments in this case.

The clinical signs in our cases were similar to PAE in humans, which seriously and negatively affected the caregivers' quality of life. Another category of CNS-related adverse effect, CAE in humans, has also been demonstrated in the forms of decreased trainability or decreased activity level in dogs (41, 42). According to the human literature, PAE associated with ASDs appeared reversible in nature (38). Further studies are warranted to provide more insights in behavior change associated with ASDs in animals.

This is the first report describing abnormal behavior episodes, similar to PAE in humans, associated with ZNS in dogs in the clinical setting.

## Data Availability Statement

The raw data supporting the conclusions of this article will be made available by the authors, without undue reservation.

## Ethics Statement

Ethical review and approval was not required for the animal study because this case report describes a medical condition of client-owned dogs without serious iatrogenic consequence. Written informed consent was obtained from the owners for the participation of their animals in this study.

## Author Contributions

SK: conception, design, and writing the draft. SK, MU, and AH: data acquisition, figure and supplemental video preparation, and revision of the draft. All authors contributed to the article and approved the submitted version.

## Conflict of Interest

The authors declare that the research was conducted in the absence of any commercial or financial relationships that could be construed as a potential conflict of interest.

## Publisher's Note

All claims expressed in this article are solely those of the authors and do not necessarily represent those of their affiliated organizations, or those of the publisher, the editors and the reviewers. Any product that may be evaluated in this article, or claim that may be made by its manufacturer, is not guaranteed or endorsed by the publisher.

## Supplementary Material

Supplementary Video 1 An abnormal behavior episode of case 1 showing aggressive growling at the family members while resting in the bed. This dog bit the client on the arm requiring the emergency room visit for deep bite wound treatment. The episode typically lasted for ~1 min.

Supplementary Video 2 An abnormal behavior episode of case 2 right after getting on the client's lap. This dog bit the client on the arm multiple times. The abrupt episode typically lasted for ~20–30 s.
